# Prevention praised, cure preferred: results of between-subjects experimental studies comparing (monetary) appreciation for preventive and curative interventions

**DOI:** 10.1186/1472-6947-13-136

**Published:** 2013-12-18

**Authors:** Ree M Meertens, Vivian MJ Van de Gaar, Maitta Spronken, Nanne K de Vries

**Affiliations:** 1 Department of Health Education and Promotion, Maastricht University, P.O. Box 616, 6200, MD Maastricht, The Netherlands; 2 Department of Public Health, Erasmus Medical Center, P.O. Box 2040, 3000, CA Rotterdam, The Netherlands; 3 Department of Social and Cultural Psychology, Radboud University Nijmegen, P.O. Box 9104, 6500, HE Nijmegen, The Netherlands

**Keywords:** Prevention, Cure, Treatment, Preference, Preference reversal, Appreciation, Urgency, Between-subjects design

## Abstract

**Background:**

'An ounce of prevention is worth a pound of cure’ is a common saying, and indeed, most health economic studies conclude that people are more willing to pay for preventive measures than for treatment activities. This may be because most health economic studies ask respondents to *compare* preventive measures with treatment, and thus prompt respondents to consider other uses of resources. However, psychological theorizing suggests that, when methods do not challenge subjects to consider other uses of resources, curative treatment is favored over prevention. Could it be that while prevention is praised, cure is preferred?

**Methods:**

In two experimental studies, we investigated, from a psychological perspective and using a between-subjects design, whether prevention or treatment is preferred and *why.* In both studies, participants first read a lung cancer prevention or treatment intervention scenario that varied on the prevention-treatment dimension, but that were the same on factors like 'costs per saved life’ and kind of disease. Then participants completed a survey measuring appreciation (general and monetary) as well as a number of potential mediating variables.

**Results:**

Both studies clearly demonstrated that, when the design was between-subjects, participants had greater (general and monetary) appreciation for treatment interventions than for preventive interventions with perceived urgency of the intervention quite consistently mediating this effect. Differences in appreciation of treatment over preventive treatment were shown to be .59 (Study 1) and .45 (Study 2) on a 5-point scale. Furthermore, participants thought that health insurance should compensate more for the treatment than for preventive measures, differences of 16% (Study 1), and 22% (Study 2). When participants were asked to directly compare both interventions on the basis of a short description, they preferred the preventive intervention.

**Conclusion:**

It appears that people claim to prefer prevention when they are asked to consider other use of resources, but otherwise they prefer treatment. This preference is related to perceived urgency. The preference for treatment may be related to the prevention-treatment dimension itself, but also to variations on other dimensions that are inherently linked to prevention and treatment (like different efficacy rates and costs per treatment).

## Background

'It is better to prevent than to cure’ is an old adage that reaches as far back as Hippocrates in Ancient Greece. This preference for prevention over treatment ensues from the idea that small preventive efforts can substantially decrease, or altogether remove, substantial negative future outcomes. This belief is not only held by lay people but by professionals as well. In fact, the National Institute for Public Health and the Environment in the Netherlands claims that, in many cases, preventive efforts can lead to great health benefits at only a small expense [1]. This praise in favor of prevention would suggest that prevention is preferred over treatment. However, the allocation of government funds suggests otherwise (although this admittedly is a very rough measure). In the Netherlands, a mere 5% of the health care budget is spent on prevention while about 40% is spent on curative measures [1]. Studies in the United States show comparable figures [2,3] despite U.S. citizens believing that the greater share of health spending should be earmarked for prevention rather than treatment of disease (1999 Harris Poll, as cited in Corso et al. [4]). This leads us to ask whether it is possible that although prevention is praised, treatment is actually preferred? Most studies comparing preferences for prevention or treatment use economic paradigms in which, for example, 'willingness to pay’ is assessed via (indirect) bidding games, trade-off questions, or discrete choice experiments. These studies indeed show a preference for prevention over treatment and, occasionally, an equally strong preference for prevention and treatment [5-10]. In almost all such studies, participants are asked to directly *compare* prevention to treatment programs or life-saving means [5-7,9,10] or the researchers go to great lengths to teach participants in tutorials to assess programs and consider other use of resources [8]. In either condition, respondents are triggered to assess programs objectively, and in doing so, they tend to show a preference for prevention because quality of life is often considered inferior after treatment.

However, in a study conducted by Corso et al. [4], participants were asked to indicate their willingness to pay for either a treatment intervention *or* a preventive intervention (both for food-borne illness with an identical reduction in mortality risk). They were not asked to compare the interventions. The results showed that respondents were far more willing to pay for treatment than for prevention. Later, Corso [11] reported data derived from an extra question completed by these same participants whereby they were asked to directly choose between a preventive or treatment intervention that were equal in terms of costs and lives saved. Interestingly, when asked to compare the two, more than 70% of the participants opted for the preventive program. Corso et al. [11] do not give explicit explanations for the observed difference between methods, although the study design is mentioned as a factor of possible influence (cf. [12]).

Evidently, in health economic studies, focus is primarily placed on what people choose when they are asked to compare the relevant facts of more interventions. In such a situation, people may see the advantages of prevention more clearly, and show a preference for prevention over treatment.

However, in everyday life, people usually hear about one intervention at a time and assessments may be less objective, and this is not only the case for lay people but also for policymakers. When the general public and policymakers would have a preference for cure in these everyday situations, this might have far-reaching consequences for policy decisions, because policymakers may be guided by their own as well as the general public’s subjective appreciations (i.e. the public opinion). With this in mind, we decided to, in the present study, focus on people’s appreciation for prevention and treatment when they are *not* asked to compare the two but rather judge one or the other independently. We believe that when asked to judge prevention or treatment independently, a number of psychological mechanisms may lead to a preference for treatment over prevention.

### Psychological mechanisms that may affect preferences for treatment and prevention

First, the time interval required for benefits to be seen may play a role. In prevention, positive outcomes tend to be long term outcomes, whereas with treatment, the positive results are often evident in the short term. Research shows that health gains that occur immediately are preferred over delayed health gains [13], cf. 'discounting’ [14,15]. It is thus plausible that treatment, with its short term results, is preferred over prevention with its long term payoff, and therefore leads to a higher measured appreciation.

Second, the certainty of the cause and effect can impact evaluations of prevention and treatment. Kahneman and Tversky [16] showed that people prefer a certain positive outcome (e.g. a sure gain of 240 dollars) over a less certain but even more positive outcome (e.g. 25% chance to gain 1000 dollars). The link between a treatment intervention and a positive outcome tends to be more evident than the relationship between a preventive activity and its positive health outcome, even if that outcome is more beneficial. As such, a more certain treatment may be valued more than uncertain prevention, and therefore may lead to a higher measured preference.

Third, the urgency of an intervention is another factor to be considered. This is influenced by the degree to which the need for intervention is tangible. With prevention, positive outcomes tend to only be evident at a collective level, whereas with treatment, success can be seen at the individual level. Because people are generally more willing to spend resources to save the life of identifiable individuals [17-21], they are probably also more likely to value programs that help identifiable people (i.e., treatment programs) rather than programs that help people that are merely represented by statistics (i.e., preventive programs). Treatment activities are therefore likely to be considered more necessary than preventive activities, especially given that bad health outcomes are usually inevitable when a person is in need of treatment [17]. This is not the case with preventive activities. In fact, at the individual level, it is impossible to predict the future development of a given health problem for which preventive activities are in place. Preventive activities may therefore seem less urgent than treatment interventions, and therefore may lead to less measured appreciation.

Fourth, the proportion of the reference group that can be 'saved’ is greater in treatment programs than it is in prevention programs. In preventive interventions, usually more individuals are treated 'in vain’ than in treatment interventions, as the number of people at risk for a disease is higher than the number of people who actually get the disease. This means that for more people the preventive intervention is a waste of time [17]. This too may lead to a preference for, and a higher measured appreciation of treatment over prevention.

Other psychological factors may play a role in preferences for health and disease related interventions, like the responsibility of the patient for the illness [7], whether the outcome is achieved actively or passively [22,23], or whether the diagnosis is benign vs. malign [23], but these factors are not consistently linked to the prevention-treatment dimension.

In short, the *time interval* required to demonstrate positive effects, the *certainty* with which the positive outcome can be *attributed* to the intervention, the extent to which the intervention is considered *urgent*, and the *proportion* of the reference group that profits from the intervention are all attributes that play a role in the desirability or appreciation of preventive and treatment interventions, and that may result in greater appreciation for activities that seek to treat rather than prevent disease. At the same time, the expected *quality of life* that results from an intervention is likely to be higher after preventive activities than after treatment activities [18]. This would suggest that prevention is valued more. According to Hsee [12] attributes that are difficult to evaluate on desirability have lower impact on preferences in separate evaluations than attributes that are easier to evaluate. We expect time interval, certainty, proportion and especially urgency to be attributes of the intervention that may be easier to evaluate than quality of life. Therefore, we expect that 'quality of life’ does not affect preference in a separate evaluation as strong as easy to evaluate attributes like 'urgency’. So, we expect that the psychological mechanisms described above generate a greater appreciation for treatment than for prevention but that this preference is only manifest when people do not have to compare prevention to treatment intervention and are not otherwise challenged to consider other use of resources. In the studies reported in this paper, we investigate if this is indeed the case and attempt to establish whether the proposed psychological mechanisms contribute to the hypothesized preference for treatment over prevention.

## Study 1

### Methods

#### Participants and design

The research design for this study was a between-subjects design with two different scenario conditions, namely a 'treatment condition’ and a 'prevention condition’. Background characteristics of the participants are described in Table 1. Chi-square tests and a t-test (age) showed that there were no significant differences in background characteristics of participants between the prevention and the treatment condition (all *p*’s > .50).

**Table 1:** Participant characteristics

		**Gender ( *n *)**		**Education ( *n *)**			**Smoking status ( *n *)**			**Age ( *M *)**
		**Male**	**Female**	**High**	**Medium**	**Low**	**Non-smoker**	**Quit smoking**	**Smoker**	
**Study 1 (*n*** **= 82)**	Prevention scenario (*n* = 43)	14	28	29	10	3	31	4	6	30.7
	Treatment scenario (*n* = 39)	15	22	29	6	2	26	5	7	31.1
**Study 2 (*n*** **= 122)**	Prevention scenario (*n* = 43)	21	19	30	9	2	25	10	6	39.6
	Treatment scenario (*n* = 79)	26	45	64	6	2	48	11	12	41.3

Note: Due to missing values *n*’s do not always sum up to totals.

#### Procedure

Data were derived from a convenience sample of train passengers traveling in the Netherlands. All available passengers in a given compartment were approached for participation and told that this study sought to assess appreciation for new developments in health care, that participation entailed completing the questionnaire that would take 10 to 15 minutes, and that they would receive a chocolate bar as a token of appreciation after completion. Potential participants were also informed that they could stop their participation in the study any time, and that they could decide not to give the researcher their questionnaire if they had any objections to what was asked of them. Only about a fifth of the train passengers declined participation. Participants who agreed to participate were randomly assigned to one of the two experimental conditions whereby they read a scenario and completed a questionnaire. The researchers stayed in the compartment while participants read the scenarios and completed their questionnaires in order to prevent people from consulting with one another. According to Dutch law, this type of questionnaire study is exempt from ethics approval. Prior to data acquisition, we pretested the questionnaires and scenarios with a pilot group of more than 50 people and small adaptations were made based on the outcomes of this pretest.

#### Scenarios

Participants first read a short introduction: 'In the following text, a description is given of a person active in the field of healthcare. Although the person does not exist in real life, this story is quite realistic. Imagine the situation as best as possible.’ A short scenario about a professor who developed a revolutionary anti-smoking intervention (in the prevention scenario) or a new surgical technique for people suffering from lung cancer (in the treatment scenario) then followed. Aside from this variation, the scenarios were kept as similar as possible (see Prevention and treatment scenario for both scenarios).

#### Prevention and treatment scenario

Prevention scenario

Under the direction of Professor Lytgens, professor in the field of preventive health care, a new revolutionary method has been developed to help people quit smoking. It concerns a course in which smokers receive an intensive training provided by a number of Professor Lytgens’ assistants over a period of two months. These assistants were trained by Professor Lytgens for half a year before being allowed to facilitate the course.

Smoking is the main cause of lung cancer. In fact, of all cases of lung cancer in the Netherlands, 90% is attributable to smoking. Less than 1% of non-smokers die because of lung cancer. The results of well-designed scientific research studies show that for every 1000 people that participate in the course, 300 stop smoking because of this course. This means that 25 people that would otherwise have died as a result of lung cancer do not. In other words, one in every 40 people that takes this course is saved. The course costs 375 euros per participant so the costs of a life saved are (40x375=) 15.000 euros (as for every 40 people, for which the costs are 375 euros per person, one life is saved)’.

Treatment scenario.

Under the direction of Professor Lytgens, professor in the field of pulmonology (lung sciences), a new revolutionary method has been developed whereby people suffering from lung cancer receive an operation that may successfully cure their cancer. Before then, an operation was pointless and these people could not be helped.

Smoking is the main cause of lung cancer. In fact, of all cases of lung cancer in the Netherlands, 90% is attributable to smoking. Less than 1% of non-smokers die because of lung cancer. The results of well-designed scientific research studies show that for every 100 lung cancer patients that would have otherwise died, 25 can be saved with the new operation. These people can live for many years in reasonably good health. In other words, one out of every four operations results in cure. Each operation costs 3750 euros so the costs of a life saved are (4x3750=) 15.000 euros (as for every four operations, each of which costs 3750 euros, one life is saved)’.

#### Questionnaires

After reading the prevention or treatment scenario, participants completed measures assessing *general* and *monetary appreciation* for the (prevention or treatment) intervention and the professor. Appreciation in this study is defined as the '*understanding of the nature or meaning or quality or magnitude of something*’ ( http://www.vocabulary.com). General appreciation was measured by eight items, monetary appreciation was measured by five items (see Concepts and corresponding questionnaire items). Participants were subsequently asked to directly compare the operation to the quitting smoking course with a simple question (see Concepts and corresponding questionnaire items 'Comparison between preventive and treatment intervention’). Measures representing concepts that may explain possible differences in appreciation between prevention and treatment (*explanatory variables*) were completed thereafter. Also, a control question that checked for differences between conditions in the degree to which the scenarios were considered to reflect real life situations (*realism of scenario)* was included. Lastly, we measured demographic characteristics. Concepts and corresponding questionnaire items shows the concepts measured, the items included in the questionnaire, and Cronbach’s alphas for the scales used. The Cronbach’s alphas for the *monetary appreciation*, *certainty of attribution,* and *urgency* scales were low (.19, .52, and .51, respectively). Low Cronbach’s alphas indicate that items measure (slightly) different constructs. So, consequently, single items were analyzed. For the General Appreciation scale, that had a sufficient to good Cronbach’s alpha, a mean score was computed (after recoding the items to 5-point scales when necessary).

#### Concepts and corresponding questionnaire items

##### ***General appreciation (Cronbach’s alpha Study 1: .73; Study 2: .82)***

How great is your appreciation for Professor Lytgens who developed the new quitting smoking course/[anti-smoking pill]/operation? 1 = very low, 10 = very high

To what extent do you appreciate Professor Lytgens’ achievement? 1 = very little, 10 = very much

Professor Lytgens should be awarded the Nobel Prize for developing the new quitting smoking course/[anti-smoking pill]/operation. 1 = disagree, 5 **=** completely agree

Professor Lytgens should get a pay raise for developing the quitting smoking course/[anti-smoking pill]/operation. 1 = disagree, 5 **=** completely agree

Professor Lytgens should get a promotion at work for developing the new quitting smoking course/[anti-smoking pill]/operation. 1 = disagree, 5 **=** completely agree

Professor Lytgens should receive attention from the media for developing the new quitting smoking course/[anti-smoking pill]/operation. 1 = disagree, 5 **=** completely agree

The new quitting smoking course/[anti-smoking pill]/operation should be prescribed internationally. 1 = disagree, 5 **=** completely agree

What grade would you give Professor Lytgens for his new quitting smoking course/[anti-smoking pill]/operation? 1 = very bad, 10 = very good

##### ***Monetary appreciation***

If a collection were to be held for work similar to that of Professor Lytgens, would you donate and, if so, how much? *(Please note: We will not ask you for money)* No, Yes, …. euro

What percentage of the costs of Professor Lytgens new quitting smoking course/[anti-smoking pill]/operation should be covered by health insurance? 0% - 10%- 20%........80% - 90% - 100%

If you could decide how health insurance funds are spent, would you give more money to Professor Lytgens’ quitting smoking course/[anti-smoking pill]/operation or to increasing personal attention to patients with incurable diseases in nursing homes? 1 = spend more money on the quitting smoking course/anti-smoking pill/operation, 5 = spend more money on nursing homes

If you could decide how health insurance funds are spent, would you give more money to Professor Lytgens’ new quitting smoking course/[anti-smoking pill]/operation or to broadcasting public service announcements (PSAs) on TV that warn people against the dangers of smoking? 1 = spend more money on the quitting smoking course/anti-smoking pill/operation, 5 = spend more money on PSAs

If you could decide how health insurance funds are spent, would you give more money to Professor Lytgens’ new quitting smoking course/[anti-smoking pill]/operation or to school-based alcohol abuse prevention activities for young people? 1 = spend more money on the quitting smoking course/anti-smoking pill/operation, 5 = spend more money on alcohol prevention

##### ***Comparison between preventive and treatment intervention***

If you could decide how health insurance funds are spent, would you give more money to Professor Lytgens’ quitting smoking course/[anti-smoking pill] (operation) or to a new operation (quitting smoking course/anti-smoking pill) that has very good results? 1 = spend more money on the quitting smoking course/anti-smoking pill (operation), 5 = spend more money on the operation (quitting smoking course/anti-smoking pill)

##### ***Explanatory variables***

Certainty of attribution

How certain are you that fewer people will die because of this quitting smoking course/[anti-smoking pill]/operation developed by Professor Lytgens? 1 = very uncertain, 5 = very certain

Professor Lytgens and his team are the persons who save people’s lives in this case. 1 = disagree, 5 **=** completely agree

Urgency

To what extent do you think it is necessary that the new quitting smoking course/[anti-smoking pill]/operation be offered to people? 1 = not necessary at all, 5 = very necessary

To what extent do you think it is necessary to develop a quitting smoking course/[anti-smoking pill]/operation that will save people that would otherwise die of lung cancer? 1 = not necessary at all, 5 = very necessary

Time interval

How long will it take before Professor Lytgens’ new quitting smoking course/[anti-smoking pill]/operation leads to a saved life? 1 = not long, 5 = very long

Quality of life

What is, in your opinion, the quality of life of the people who were saved by Professor Lytgens’ new quitting smoking course/[anti-smoking pill]/operation? 1 = very bad, 5 = very good

Proportion of the reference group who profit

Many people who are treated with Professor Lytgens’ new quitting smoking course/[anti-smoking pill]/operation are treated in vain. 1 = completely disagree, 5 = completely agree

##### ***Control question***

To what extent do you find the scenario about Professor Lytgens realistic? 1 = very unrealistic 5 = very realistic

##### ***Demographic characteristics***

Are you male or female? Male - Female

What is your age? ..................

What is the highest level of education you have completed? less than high school - high school – lower vocational training – higher vocational training/college - Bachelor’s degree at a university - Master’s degree or more at a university

##### ***Smoking habits***

Do you smoke? Yes - No - I quit smoking

## Results

A MANOVA was executed with scenario condition as the independent variable and the appreciation measures as dependent variables (see Table 2). The results showed participants to have significantly greater general appreciation for the treatment intervention than for the preventive intervention, a difference of .59 on a 5-point scale. Monetary appreciation was also greater in the treatment scenario condition. Participants in the treatment condition reported a significantly higher percentage (*M* = 63%) than those in the prevention condition (*M* = 47%) when asked how much of Lytgens’ intervention should be covered by health insurance. At the same time, participants in the prevention condition were *more* inclined to allocate money to Lytgens’ intervention than to an alternative smoking prevention intervention, namely an anti-smoking public service announcement (PSA), than participants in the treatment condition.

**Table 2:** Multivariate and univariate effects of scenario condition on appreciation measures, means, standard deviations and range (Study 1)

	**Range**	***Df***	***F***	***p***	***η***_***p***_^***2***^	***M***_***prev. ***_ ***(SD)***	***M***_***cure ***_ ***(SD)***
Multivariate		6.66	6.04	0.000	.36		
Univariate							
General appreciation scale	1-5	1.71	26.25	0.000	.27	3.18 (.48)	3.77 (.50)
Intention to donate money (amount)	0-..	1.71	1.52	n.s.	.02	1.11 (2.18)	4.51 (16.92)
Compensation by health insurance	1-10	1.71	5.86	0.02	.08	4.71 (2.42)	6.31 (3.22)
Allocation of money in Lytgens’ intervention/nursing homes	1-5	1.71	<1	n.s.	.001	2.24 (1.15)	2.31 (.96)
Allocation of money in Lytgens’ intervention/anti-smoking PSAs	1-5	1.71	4.32	0.04	.06	3.29 (1.21)	2.66 (1.39)
Allocation of money in Lytgens’ intervention/alcohol prevention	1-5	1.71	1.40	n.s.	.02	2.42 (1.03)	2.74 (1.29)

On the variable *operation versus quitting smoking course* (see Concepts and corresponding questionnaire items under ’Comparison between preventive and treatment intervention’), data were recoded such that, for both questionnaires, a score of 1 indicated a preference for prevention and a score of 5 a preference for treatment. A one-sample T-test with 3 as a test value on the recoded item that directly compared the course (prevention) to the operation (treatment) revealed a preference for the former, *t*(80) = -2.16, *p* = 0.03 (*M* = 2.68, *SD* = 1.35).

### Mediation analyses

In accordance with the procedures suggested by Baron and Kenny [24], we investigated if any of the explanatory variables mediated the effect of the scenario condition on appreciation measures. For this to be the case, [1] the scenario condition must account for significant variance in the appreciation measure; [2] the scenario condition must yield significant variance in the explanatory variable; [3] the explanatory variable must, while controlling for the scenario condition, produce significant variance in the appreciation measure; and, at the same time, [4] the significance of the association between the scenario condition and the appreciation measure must decline. We thus first conducted six regression analyses with the scenario condition as the predictor and the appreciation measures as the dependent variables (step 1). We then conducted seven separate regression analyses with the scenario condition as the predictor variable and explanatory variables (certainty of attribution to intervention [2 items], urgency of intervention [2 items], time interval for positive effects to manifest, proportion of the reference group that profits, and quality of life) as dependent variables (step 2). In the third step, separate linear regression analyses were run with the explanatory variables that were significant in step 2 and scenario condition as predictors, and the appreciation measures that came out of step 1 as dependent variables.

Table 3 shows that the criteria for step 1 were fulfilled only by the *general appreciation* scale, the item on the *percentage health insurance should compensate,* and the item in which participants indicated the degree to which *money should be allocated to Lytgens’ intervention or to anti-smoking PSAs.* The table also shows that the criteria for step 2 were met by the items *urgency to introduce the method* and *certainty of attribution ('Professor Lytgens is the person who saves lives’)*. In the step 3 analyses, we explored the effects of the significant explanatory variables (*urgency to introduce method* and *certainty of attribution*) on the three appreciation items significant in step 1 (*general appreciation*, *percentage that health insurance should compensate*, *money allocation to intervention or PSAs*) while controlling for the scenario condition. Significant results were found for the *urgency to introduce the method* on *general appreciation* and on the *percentage health insurance should compensate*. On both appreciation measures, the influence of the scenario condition reduced, but the test for mediation was significant only for *general appreciation*, *Sobel Z* = 2.70, *p* < .01. In short, these findings show that the effect of scenario condition on general appreciation is partially mediated by the perceived urgency to introduce the proposed method.

**Table 3:** Significant effects of the mediation analyses (Study 1)

**Step 1: Effect of scenario condition on appreciation measures**	
General appreciation scale	*B* = .60, *SE* = .11, *p* < .001
Percentage that health insurance should compensate	*B* = .17, *SE* = .63, *p* = .01
Allocation of money to Lytgens’ intervention/PSAs	*B* = .60, *SE* = .30, *p* = .05
**Step 2: Effect of scenario condition on explanatory variables**	
Urgency to introduce method	*B* = .61, *SE* = .18, *p* = .001
Certainty of attribution	
(Professor Lytgens is the person who saves lives)	*B* = .77, *SE* = .23, *p* = .001
**Step 3: Effect of significant explanatory variables on significant appreciation measures while controlling for scenario condition**	
General appreciation scale	
-Urgency to introduce the method	*B* = .29, *SE* = .07, *p* < .001
Percentage that health insurance should compensate	
-Urgency to introduce the method	*B* = 2.06, *SE* = .33, *p* < .001
**Step 4: Effect of scenario condition on significant appreciation measures while controlling for significant explanatory variables**	
General appreciation scale	*B* = .41, *SE* = .12, *p* = .001
Percentage that health insurance should compensate	*B* = .32, *SE* = .58, *p* = n.s.

### Realism of scenarios and smoking behavior of participants

The extent to which the scenarios were perceived as realistic could have influenced the differences found between the two scenario conditions, as could the smoking behavior of the participants. To test this, we first conducted a univariate analysis with scenario condition as the independent variable and realism of scenarios as the dependent variable. No significant difference was found between the two conditions, *F*(1,77) = 0.86, *p* = 0.36. We then conducted a MANOVA with scenario condition and smoking behavior as independent variables, and general appreciation, the amount of money participants would donate, percentage that health insurance should compensate, and three questions on how health insurance should invest funds (intervention versus increasing personal attention in nursing homes, PSAs, or school-based alcohol abuse prevention). No main multivariate effect of smoking behavior was found nor was a multivariate interaction effect of scenario condition with smoking behavior found (*p*s > .49).

## Discussion Study 1 and introduction Study 2

This first study demonstrated more general and monetary appreciation for a treatment intervention than for a preventive intervention when participants could not directly compare the interventions. Participants rating the treatment scenario reported a substantial greater percentage when asked how much of the intervention should be covered by health insurance. One exception to the general finding that treatment is preferred was that, compared to participants in the treatment condition, participants in the preventive condition conveyed a willingness to allocate a relatively greater sum to the proposed preventive intervention than to anti-smoking PSAs. Perhaps participants in the prevention condition reasoned that the evidence for the proposed preventive intervention of a quitting smoking course was stronger than for anti-smoking PSAs, which is in fact just another preventive activity. In contrast, participants in the treatment condition, who were not told about the effective smoking prevention course, may have reasoned that at least some money should go to smoking prevention, especially given the fact that the expenses of the proposed lung cancer operation are high.

The mediation analyses suggested that only the urgency to introduce the proposed method plays a role in explaining differences in appreciation between prevention and treatment. This is in accordance with earlier research showing a preference for helping more severely ill patients [25]. Quite logically, the need to intervene is perceived as greater when someone is already ill than when someone is merely at risk for a disease. The other explanatory variables for either a preference for treatment (i.e., time interval for positive effects, and proportion that profits) or a preference for prevention (i.e., quality of life) did not vary between the two scenario conditions, except for one of the 'certainty of attribution’ items.

As expected, the preference for treatment disappeared when participants were asked to directly compare prevention to treatment interventions. However, it must be noted that this comparison was made on basis of a simple question only, with incomplete information about the option not presented in the scenario (Concepts and corresponding questionnaire items 'Comparison between preventive and treatment intervention’). In fact, the preventive intervention was valued significantly more than the treatment intervention and additional analyses showed that appreciation for the preventive intervention was actually significantly higher among participants in the treatment condition (*F*(1,76) = 7.82, *p* = 0.01 with *M*_prevention_ = 3.36, *SD* = 1.16 and *M*_treatment_ = 4.08, *SD* = 1.11). This suggests that when people have to choose between a preventive and a treatment intervention, they prefer prevention more when they just were presented with the costs and complexities of a treatment intervention.

Clearly, as previous research has demonstrated, most people, when allowed to compare, maintain that prevention is better than treatment but when they are merely presented with one or the other, which is often the case in real-life situations, treatment is preferred. Given that preference for prevention is greatest when presented alongside a treatment intervention, we contend that preference for prevention can be stimulated by providing information about the costs and complexities of treatment interventions and outlining that these cost and complexities of treatment can be prevented.

Although the findings of this study support the idea that treatment is preferred to prevention in a between-subjects design, caution should be applied when generalizing these results. Many preventive methods exist and this study only presented a quitting smoking course, which is an intervention rooted in behavioral science. Because the treatment (an operation) was an intervention rooted in medical science, differences in appreciation might also be attributed to a different appreciation of these sciences. So it is possible that, had our prevention scenario described a preventive intervention rooted in medical rather than behavioral science (e.g., drug treatment), other results could have been produced. As a result, our second study replicates the first, but rather than proposing a quitting smoking course as the prevention intervention, it proposes prevention through an anti-smoking pill.

## Study 2

### Methods

#### Participants and design

Similarly to Study 1, the research design was between-subjects with two scenario conditions, namely a 'treatment condition’ and a 'prevention condition’. Background characteristics of participants are presented in Table 1. Chi-square tests and a t-test (for Age) showed there were no significant differences in background characteristics between participants of Study 1 and Study 2 (all *p*’s > .25), and also no significant differences in background characteristics of participants between the prevention and the treatment condition (all p’s > .09).

#### Procedure

The procedure, scenarios, questionnaires, and data analyses were identical to those of Study 1 with two exceptions. First, the questionnaires were not completed by train passengers but rather online by participants derived from a database of people who had previously volunteered to participate in questionnaire studies. Second, the scenario for the preventive intervention did not describe a quitting smoking course but rather an 'anti-smoking pill’ with identical effectiveness and costs as described in Study 1’s prevention scenario. Cronbach’s alpha of the general appreciation scale was good (see Concepts and corresponding questionnaire items) but for the monetary appreciation items, the certainty of attribution items, and the urgency of intervention items, Cronbach’s alphas were, once again, low (.17, .44, and .55, respectively). We therefore analyzed the items separately, as we did in Study 1.

## Results

A MANOVA was executed with scenario condition as the independent variable and the appreciation measures as dependent variables (see Table 4). The results showed participants to have significantly greater general appreciation for the treatment intervention than for the preventive intervention, a difference of .45 on a 5-point scale. Monetary appreciation was also greater in the treatment scenario condition. Participants in the treatment condition reported a significantly higher percentage (78%) than those in the prevention condition (56%) when asked how much of Lytgens’ intervention should be covered by health insurance. Participants in the prevention condition, compared to the treatment condition, were less inclined to allocate money to Lytgens’ intervention than to other prevention interventions, namely to increasing personal attention for people with incurable diseases in nursing homes and alcohol abuse prevention in schools. No univariate effects were found for the intention to donate money or for the item that asked participants the degree to which they would rather invest in Lytgens’ intervention than in anti-smoking PSAs. It is important to note that the non-parametric test performed to check the results of the analyses of variance (see endnote ^a^) showed a higher intention to donate money in the treatment condition than in the preventive condition, *U* = 1202.00, *p* = .01; Mean rank_prevention_ = 49.95; Mean rank_treatment_ = 63.53.

**Table 4:** Multivariate and univariate effects of scenario condition on appreciation measures in Study 2

	***Range***	***Df***	***F***	***p***	***η***_***p***_^***2***^	***M***_***prev. ***_ ***(SD)***	***M***_***treat***_ ***(SD)***
Multivariate		6.104	6.26	0.000	.27		
Univariate							
General appreciation scale	1-5	1.109	16.17	0.000	.13	3.31 (.66)	3.76 ( .50)
Intention to donate money (amount)	0-..	1.109	<1	n.s.	.000	4.34 (17.32)	4.62 (12.91)
Compensation by health insurance	1-10	1.109	17.27	0.000	.14	5.62 (2.90)	7.82 (2.53)
Allocation of money in Lytgens’ intervention/nursing homes	1-5	1.109	14.76	0.000	.12	3.93 (.89)	3.15 (1.08)
Allocation of money in Lytgens’ intervention/anti-smoking PSAs	1-5	1.109	1.07	n.s.	.01	3.05 (1.18)	2.79 (1.33)
Allocation of money in Lytgens’ intervention/alcohol prevention	1-5	1.109	6.2	0.01	.05	3.70 (1.02)	3.14 (1.19)

A one-sample T-test with 3 as a test value on the recoded item that compared the anti-smoking pill to the operation (see Concepts and corresponding questionnaire items 'Comparison between preventive and treatment intervention’) revealed a preference for the preventive intervention, *t*(112) = -3.61, *p* < 0.001 (*M* = 2.55, *SD* = 1.33).

### Mediation analyses

Similar to Study 1, we conducted mediation analyses in accordance with Baron and Kenny [24]. Table 5 shows that the criteria for Step 1 were fulfilled by the *general appreciation* scale, the item on *percentage health insurance should compensate,* the items in which participants indicated the degree to which *money should be allocated to Lytgens’ intervention or to increasing personal care in nursing homes* or to *alcohol abuse prevention in schools.* The table further shows that the criteria for Step 2 were met by both *urgency of intervention* items (*urgency to introduce the method* and *urgency to develop similar methods*), both *certainty of attribution* items *('Professor Lytgens is the person who saves lives’* and *'How certain are you that less people die of lung cancer because of Professor Lytgen’s pill/operation?*’*),* and the *time interval*. In short, participants were more convinced that the treatment intervention is urgent, certain, and has positive effects in the short term.

**Table 5:** Significant effects of the mediation analyses (Study 2)

**Step 1: Effect of scenario condition on appreciation measures**	
General appreciation scale	*B* = .44, *SE* = .11, *p* < .001
Percentage that health insurance should compensate	*B* = 2.20, *SE* = .52, *p* < .001
Allocation of money to Lytgens’ intervention/nursing home	*B* = -.75, *SE* = .20, *p* < .001
Allocation of money to Lytgens’ intervention/alcohol prevention	*B* = -.56, *SE* = .22, *p* = .01
**Step 2: Effect of scenario condition on explanatory variables**	
Urgency to introduce method	*B* = .48, *SE* = .15, *p* = .002
Urgency to develop similar methods	*B* = .31, *SE* = .15, *p* = .04
Certainty of attribution (Professor Lytgens saves lives)	*B* = .84, *SE* = .19, *p* < .001
Certainty of attribution (less mortality)	*B* = .42, *SE* = .16, *p* = .01.
Time interval	*B* = -.59, *SE* = .18, *p* = .002
**Step 3: Effect of significant explanatory variables on significant appreciation measures while controlled for scenario condition**	
General appreciation scale	
-Urgency to introduce the method	*B* = .16, *SE* = .07, *p* = .03
-Certainty of less mortality	*B* = .20, *SE* = .06, *p* = .002
Percentage that health insurance should compensate	
-Urgency to introduce the method	*B* = 1.53, *SE* = .37, *p* < .001
Allocation of money to Lytgens’ intervention/nursing homes	
-Urgency to introduce the method	*B* = -.34, *SE* = .15, *p* = .03
**Step 4: Effect of scenario condition on significant appreciation measures while controlling significant explanatory variables**	
General appreciation scale	*B* = .16, *SE* = .10, *p* = .13
Percentage that health insurance should compensate	*B* = 1.39, *SE* = .54, *p* = .01
Allocation of money to Lytgens’ intervention/ nursing homes	*B* = 1.39, *SE* = .54, *p* = .01

In the step 3 analyses, we explored the effects of the five explanatory variables significant in step 2 on the four appreciation scales/items significant in step 1 while controlling for scenario condition. Significant results were found for *urgency* to introduce the method and *certainty* that mortality will decrease on *general appreciation*. In fact, *urgency to introduce the method* was also significant for *percentage health insurance should compensate* and for the degree to which *money should be allocated to Lytgens’ intervention or to nursing homes*. On all three appreciation measures, the influence of scenario condition reduced. Separate Sobel tests showed this reduction to be significant both for *urgency to introduce the method*, *Z* = 2.76, *p* < .001, and *certainty that mortality will decrease*, *Z* = 2.25, *p* < .05 on *general appreciation*. Also, on the items *percentage health insurance should compensate* and *money should be allocated to Lytgens’ intervention or to nursing homes*, Sobel tests showed the reduction to be significant for *urgency to introduce the method*, *Z* = 2.77, *p* = .01 and *Z* = -2.39, *p* = .02, respectively. In short, the mediation analyses showed that the effect of scenario condition on three appreciation scales is partly mediated by the urgency to introduce the method and that the effect of scenario condition on general appreciation is also mediated by the certainty that less people will die of lung cancer because of the intervention.

### Realism of scenarios and smoking behavior of participants

As was the case in Study 1, a univariate analysis was executed with the scenario condition as the independent variable and the realism of scenarios as the dependent variable. No significant difference was found between the two scenario conditions, *F*(1,110) = 3.17, *p* = 0.08. However, given the p-value, we conducted a MANCOVA with scenario condition as an independent variable, perceived realism of the scenario as a covariate, and general appreciation, the amount of money participants would donate, percentage that health insurance should compensate, and the three questions on how health insurance should invest funds as dependent variables. The multivariate regression of the covariate was not significant (*p* = .32). We then conducted a MANOVA with scenario condition and smoking behavior as independent variables and the same dependent variables used in the MANCOVA. No main multivariate effect of smoking behavior was found nor was a multivariate interaction effect of scenario condition with smoking behavior found (*p*s > .20).

## Discussion

The results of the two studies presented in this paper challenge the old adage 'an ounce of prevention is worth a pound of cure.’ Both studies showed a preference for treatment over prevention when participants do not directly compare one to the other. The main hypothesis was thus confirmed. On the whole, more general and monetary appreciation was shown for a treatment intervention than for a preventive intervention that was analogous in terms of the disease in question, the information on the most important risk factor (smoking), and the costs per saved life.

In the first study, the scientific domain of the interventions may have differed with the quitting smoking course being perceived as a behavioral intervention and the operation being seen as a medical intervention. Differences in appreciation for these two fields of science could have led to the differences in appreciation for the proposed preventive and treatment interventions. However, in our second study, both interventions were medical interventions and still the treatment condition was preferred to prevention.

We thus conclude that this significantly stronger appreciation for treatment is quite robust. At the same time, we strongly suggest replicating this study with other diseases and with other study populations. It also appears that the preference for treatment is limited to situations in which one cannot or chooses not to compare preventive and treatment interventions. In both studies, when participants compared the intervention they initially read about in the scenario with (a short description of) the other intervention, thus when they were challenged to consider other use of resources, their preference shifted to prevention. This is in line with Corso et al. [4]. Preference for prevention in these instances appears to be a matter of momentarily paying lip service to prevention. This is not to say that preference for treatment, as found in our study, is a better reflection of reality or that health economic studies should not use comparative study designs. The present study merely shows that there are psychological forces at work that undermine the preference for prevention most people proclaim when challenged to comparatively assess prevention and treatment and this is important to recognize as appreciation for prevention or treatment may have far-reaching consequences for policy-making in general and health budget allocation in particular.

In our studies, we also endeavored to gain insight regarding why people prefer treatment to prevention. Several potential explanations based on a number of proposed psychological mechanisms were tested. We explored whether preference for treatment was related to the fact that treatment has more short term effects, greater certainty with regard to the attribution of positive outcomes, a higher proportion of people who profit, and greater perceived urgency. Following Hsee et al. [12], we expected that easy to evaluate attributes like urgency would have a stronger impact on appreciation than more difficult to evaluate attributes like quality of life. Indeed, we found that the urgency of the intervention and particularly the urgency to introduce the proposed intervention (operation, quitting smoking course, or anti-smoking pill) most consistently explained the preference for treatment over prevention. In fact, urgency mediated the effect of the type of intervention on general appreciation in both the first and the second study in addition to mediating the effect of the type of intervention on two monetary appreciation items in the second study. The importance of urgency in explaining differential appreciation for prevention and treatment corresponds with the idea that people are more willing to spend resources on identifiable individuals than on people who are just statistics [16]. Concrete and identifiable individuals may be expected to raise more vivid images and more emotions [26] than 'statistic’ people. These findings are also in line with the Construal-level Theory of Psychological Distance [27]: the time delay associated with prevention and the hypothetical nature of it (two dimensions that according to this theory promote psychological distance) may lead to a relatively abstract construal of prevention measures compared to treatment (that is relatively concrete), and also to more perceived social distance. The higher perceived social distance may lead to a low perceived urgency of prevention measures compared to treatment. An interesting line for future research would be to further investigate whether differences in perceived psychological distance indeed lead to differences in appreciation between prevention and treatment.

Furthermore, our finding that the certainty that an intervention leads to less mortality mediates the effect of the type of intervention on general appreciation, as found in our second study, supports for the work of Tversky and Kahneman [15] who claim that people prefer relatively certain positive outcomes to less certain but potentially more positive outcomes.

In exploring what drives preference for treatment to prevention, it is important that we not only look at the variables that predict this preference but also the variables that do not predict the preference for treatment over prevention. Our findings showed no support for the contention that the time interval required for positive outcomes to manifest and the proportion of the target group that profits impact preferences for treatment over prevention. This was surprising given that Read and Read [13] found a preference for positive outcomes in the short term and Jenni and Loewenstein [17] found that the difference in proportion between statistical and identifiable victims was the main cause for the identifiable victim effect. However, one possibility is that at least some of the respondents who read the preventive scenario understood the question about the proportion of people treated in vain to mean the proportion people that did not stop smoking (instead of the proportion that did not profit from the intervention in terms of dying of lung cancer). Moreover, in our study, we found that quality of life after the intervention was similar for both the prevention and the treatment conditions. Could it be that a life without smoking is not considered a more positive outcome than a life after an operation?

One could argue that a limitation of both studies is that the variation on the prevention-treatment dimension went hand in hand with variations on other dimensions. For example, although the costs of a saved life were the same in both interventions, the costs of treatment (375 vs. 3750 euros), the relative efficacy (1/40 vs. ¼) and the nature of the intervention (training or pill vs. surgery) differed. Therefore, we cannot exclude the possibility that the preference for treatment was not caused by a difference in preference for treatment and prevention per se, but by differences on these dimensions. The relative influence of these factors could be disentangled in follow-up studies. However, we would argue that in real life these differences are inherent to differences between prevention and treatment: prevention and treatment in general have different methods, and because prevention is almost by definition directed at a larger population than treatment, efficacy tends to be lower and so invested money per person should also be lower to lead to equal costs of a saved life.

## Conclusions

The results of our study have shown that when asked to compare and so are triggered to consider other use of resources, people prefer prevention but when subjective assessments get a chance, treatment is clearly preferred. In these instances, treatment is appreciated more in general and also in terms of how funds should be allocated. The preference for treatment may be related to the prevention-treatment dimension itself, but also to variations on other dimensions that are inherently linked to prevention and treatment (like different efficacy rates and costs per treatment). The greater appreciation for treatment appears to be related to the perceived urgency of the intervention. Of course urgency is greatest when it is almost too late.

## Endnote

^a^As assumptions of homogeneity and normality were, in some instances, violated, we used Mann–Whitney U-tests to check the results on the appreciation measures. These findings are only reported when they diverged from the other analyses (which was only the case on one variable in Study 2).

## Competing interests

The authors declare that they have no competing interests.

## Authors’ contributions

RM conceived the studies, contributed to the data analysis and drafted the manuscript. VdG participated in the design of study 2, and collected the data of this study, and wrote a first draft of study 2. MS participated in the design of study 1, collected the data of this study, and wrote a first draft about this part of the research. NdV participated in design of the studies and in drafting the manuscript. All authors read and approved the final text.

## Pre-publication history

The pre-publication history for this paper can be accessed here:

http://www.biomedcentral.com/1472-6947/13/136/prepub
